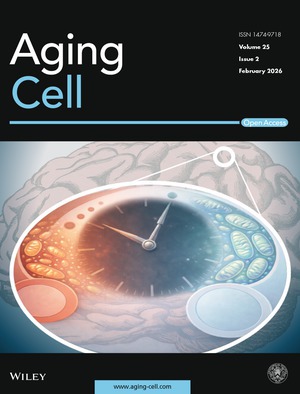# Additional Cover

**DOI:** 10.1111/acel.70412

**Published:** 2026-02-15

**Authors:** Madison Milan, Eva Troyano‐Rodriguez, Jennifer Ihuoma, Sharon Negri, Rakesh Rudraboina, Aleksandra Kosmider, Shantipriya Awasthi, Priya Balasubramanian, Shannon Conley, Andriy Yabluchanskiy, Anna Csiszar, Zoltan Ungvari, Rafael de Cabo, Stefano Tarantini

## Abstract

The cover image is based on the article *Fasting as Medicine: Mitochondrial and Endothelial Rejuvenation in Vascular Aging* by Madison Milan et al., https://doi.org/10.1111/acel.70372.